# Development of a Cost-Effective and Low-Toxicity Safranin-Based Assay for Discovering *Candida albicans* Biofilm Formation Inhibitors in Large-Scale Screening Campaigns

**DOI:** 10.3390/jof12080581

**Published:** 2026-08-07

**Authors:** Augusto Vazquez-Rodriguez, Jose L. Lopez-Ribot

**Affiliations:** Department of Molecular Microbiology and Immunology and South Texas Center for Emerging Infectious Diseases, The University of Texas at San Antonio, San Antonio, TX 78259, USA; jesus.vazquezrodriguez@utsa.edu

**Keywords:** *C. albicans*, biofilm, antifungal drugs, high-throughput screening

## Abstract

Screening assays for discovering *Candida albicans* biofilm inhibitors typically measure either metabolic activity or attached biomass. Although biomass staining with Crystal Violet or Safranin is affordable and easy to perform, standard protocols often require toxic solvents for biofilm fixation and dye extraction. We developed and evaluated an alternative Safranin staining method that uses heat fixation and direct absorbance measurement of stained biofilms without a dye-extraction step. Under the experimental conditions tested, the proposed method reduced solvent use and plate handling and minimized reagent use while offering assay consistency and reliability for primary screening campaigns. The assay quality was verified using positive and negative inhibition controls, with acceptable Z′-factor values supporting its use for primary drug screening. The protocol was further evaluated by screening 1520 compounds from the Prestwick Chemical Library to identify inhibitors of *C. albicans* biofilm formation. The proposed Safranin method provides a simplified, extraction-free alternative for biofilm-inhibitor screening under the experimental conditions evaluated in this study.

## 1. Introduction

Typically, large-scale drug screening protocols for identifying *C. albicans* biofilm formation inhibitors rely on measuring either the metabolic activity or the biomass of attached biofilms at the bottom of microtiter plate wells. For biofilm metabolic activity assays, Formazan-based compounds (e.g., XTT, MTT, and WST-1) and Resazurin-based compounds (e.g., Resazurin, Alamar Blue, Presto Blue) have been widely used, while most studies rely on Crystal Violet and Safranin staining for biofilm biomass quantification [1,2,3,4,5]. An ideal scenario for antifungal drug screening campaigns would be to test potential drugs using orthogonal assays; however, given constraints on experimental processing times, equipment limitations, and the cost of purchasing the necessary reagents, this strategy is not always feasible for all laboratories worldwide. In settings where reagent costs and equipment availability are limiting, biofilm staining methods may provide a practical option because they can be performed with commonly available microplate reader equipment and relatively inexpensive staining reagents. Nonetheless, Crystal Violet has been reported to be toxic, and although Safranin is a low-toxicity dye, both protocols require the use of toxic solvents (mainly pure ethanol, methanol, and acetic acid) for fixing biofilms and extracting bound dye for subsequent absorbance measurements [6,7,8,9].

Considering these factors, we aimed to provide an alternative use of the Safranin staining protocol, given its low toxicity and cost-effectiveness. The use of organic solvents for biofilm fixation was replaced with heat fixation. We also avoided solvent extraction of Safranin by directly measuring the optical density (OD) of Safranin-stained biofilm biomass. This single modification eliminated the need for solvents to extract Safranin while reducing the number of microtiter plates required for OD readings. We adapted this method for its use in 96-well and 384-well plate formats. The uniformity and reproducibility of the assay were evaluated and finally validated by screening the Prestwick Chemicals 1520 drug library to identify biofilm-inhibiting compounds.

## 2. Materials and Methods

### 2.1. Fungal Cultures

For this study, we used the *C. albicans* strain SC5314, a robust biofilm-forming strain widely used as a standard for *C. albicans* biofilm and molecular research [1,10,11]. Fresh agar stocks (prepared from frozen glycerol stocks) were used to inoculate yeast extract–peptone–dextrose (YPD) broth medium (1% [*w*/*v*] yeast extract, 2% [*w*/*v*] peptone, 2% [*w*/*v*] dextrose). Cultures were incubated overnight at 30 °C with agitation at 150 rpm. After incubation, the cultures were washed twice with phosphate-buffered saline (PBS), and the resulting cell pellet was resuspended again in PBS. The cell concentration was measured using a disposable counting chamber. To prepare the working inoculum, cells were diluted in RPMI-1640 medium (without sodium bicarbonate, supplemented with L-glutamine, buffered with 165 mM morpholinepropanesulfonic acid, and adjusted to pH 6.9) to the desired cell concentration, as determined by the experimental conditions.

### 2.2. Compounds and Reagents

The Drug Library was obtained from Prestwick Chemical (San Diego, CA, USA) and comprised 1520 off-patent small molecules. The Drug Library consisted of a set of 96-well microtiter plates containing compound solutions in DMSO (at 10 mM). Intermediate stock solutions were then prepared in 96-well microtiter plates by diluting 10 mM solutions down to 1 mM with DMSO. Finally, “drug screening” plates were prepared by transferring 2 µL of each compound solution (1 mM) into tissue-treated flat-bottom 96-well plates. These plates were then sealed with plastic storage film and stored at −20 °C until use. Amphotericin B (AmB) solution (250 µg/mL) and Niclosamide (NIC) were procured from Sigma-Aldrich (St. Louis, MO, USA). AmB was used as a positive control for inhibition in all the assays performed in this study. Safranin-O (1% *w*/*v*) for biofilm staining was procured from Aldon Chemicals (Avon, NY, USA).

### 2.3. Safranin Staining Optimization for C. albicans Biofilms

First, we explored the optimal Safranin concentrations for staining attached biofilms. To grow uniform and homogeneous individual *C. albicans* biofilms on 96-well plates, we followed the conditions outlined in a well-established protocol [1]. Working inoculum (1 × 10^6^ cells/mL) was prepared as previously mentioned in RPMI-1640 medium, and 96-well plates were seeded with either 100 μL of the inoculum (positive growth controls) or RPMI-1640 medium (background controls) into alternating rows. The plates were sealed with a gas-permeable film to prevent evaporation and minimize variability and incubated at 37 °C for 24 h under static conditions. After incubation, the biofilms in the wells were washed twice with PBS, and the adherent biofilm biomass was heat-fixed to the bottom of the wells at 65 °C for 30 min. Positive growth and background control wells were then stained with 100 µL of aqueous Safranin solutions at decreasing concentrations (1%, 0.5%, 0.25%, and 0.1% *w*/*v*) for 30 min. The stained biofilms were then washed twice with bidistilled water to remove excess dye, dried at 65 °C for 30 min, and the optical density at 490 nm (OD_490_) was measured directly from the Safranin-stained biofilms. A one-way ANOVA with Tukey’s post hoc test was used to evaluate differences in staining efficiency. Signal-to-background (S/B) ratios and coefficients of variation (CoVs) were also calculated [12].

### 2.4. Quality Assessment and Reproducibility of the 96-Well Plate Assay

After optimizing staining conditions, we assessed signal uniformity in Safranin-stained biofilms across the 96-well plate format. Inoculum was prepared as described earlier in RPMI-1640 (1 × 10^6^ cells/mL), and 100 µL was used to seed entire 96-well plates into non-treated wells (negative controls for inhibition), while wells treated with AmB (5 µg/mL) served as positive inhibition controls. Separate full plates (96 wells each) were used for the positive and negative controls for each experimental run. Plates were stained with Safranin (0.1% *w*/*v*) and processed as mentioned previously. OD_490_ readings from control wells were used to calculate the Z′-factor, evaluating the uniformity and reliability of the protocol [13,14]. The Z′ factor is a dimensionless statistical value used to measure the quality and robustness of large-scale screening assays. It evaluates both the signal dynamic range and data variation using positive and negative controls, with a score above 0.5 indicating an excellent, reliable assay. It provides a useful tool for comparison and evaluation of the overall quality of assays and has been utilized extensively in both assay optimization and validation [12,14]. Next, to assess the reproducibility of the protocol, we performed a dose–response assay using two-fold serial dilutions of AmB (10–0.078 µg/mL), adding 50 µL of each dilution and 50 µL of working inoculum (2 × 10^6^ cells/mL) per well, halving both cell and drug concentrations. Plates were then sealed with gas-permeable film and incubated for 24 h at 37 °C. Afterward, the plates were stained with Safranin (0.1% *w*/*v*) and processed as previously described. OD_490_ values were normalized to those of the control wells, and the percentage of inhibition was calculated as previously described [15]. IC_50_ values were determined by fitting normalized data to the variable-slope Hill equation using GraphPad Prism (version 10.4.1).

### 2.5. Validation of the 96-Well Plate Assay and Large-Scale Screening of the Prestwick Chemical 1520 Library

As an experimental validation, we evaluated the ability of the assay to identify positive biofilm-inhibitory compounds by simulating a primary drug screening campaign. “Mock” plates included positive and negative controls for inhibition and NIC (20 µM), a previously described biofilm inhibitor [16,17]. Inoculum (1 × 10^6^ cells/mL in RPMI-1640) was used to seed the wells, obtaining a final vol. of 100 µL/well, and 96-well plates were incubated at 37 °C for 24 h. After incubation, the plates were rinsed with PBS, stained with Safranin (0.1% *w*/*v*), and processed as described in the previous section. The percentage of inhibition and Z′-factors were calculated as previously described [12,13].

Following validation, the Prestwick Chemical 1520 chemical library was screened to identify biofilm-formation inhibitors using our proposed Safranin staining protocol. Briefly, drug screening plates were seeded with inoculum (1 × 10^6^ cells/mL in RPMI-1640), resulting in a final volume of 100 µL per well. AmB (5 µg/mL) and non-treated wells served as positive and negative controls for inhibition, respectively. Plates were incubated and processed for Safranin staining as described in the previous sections. OD_490_ values were measured and normalized to the control wells, and the percentage of inhibition was correspondingly calculated. Primary hits were defined as molecules that showed >80% inhibition and had no previously reported antifungal activity. Finally, the inhibitory activity of the potential hits was confirmed through a dose–response assay, performed as described in the previous sections.

### 2.6. Adaptation, Quality Assessment, and Validation of the Safranin-Based Protocol for 384-Well Plate Format

To adapt the multi-well plate protocol for application in a 384-well tissue-treated flat-bottom plate (384-well plate), optimal conditions from the 96-well plate format were extrapolated and validated. First, we assessed the robustness and quality of the assay experimentally by calculating the Z′-factor. Briefly, *C. albicans* inoculum was prepared in RPMI-1640 (1 × 10^6^ cells/mL), and 50 µL was dispensed into alternating rows of the same plate. Positive and negative controls for inhibition were arranged in full alternating rows (16 wells) in the same 384-well plate, with either treatment (AmB at 5 µg/mL) or non-treated wells. Each control had 192 dedicated wells within the same plate for the experimental run. Plates were then sealed with gas-permeable film and incubated at 37 °C for 24 h. After incubation, the supernatant was removed, and the wells were rinsed twice with PBS and then dried at 65 °C for 30 min. After the first drying, biofilms in the wells were stained with 50 µL of safranin solution (0.1% *w*/*v*) and incubated for 30 min at room temperature. Following staining, wells were rinsed twice with PBS to remove excess stain, and biofilms were dried at 65 °C for 30 min. After drying, the OD_490_ of the stained biofilms was directly measured from the plates. Absorbance readings were then used to calculate the Z′ score statistics.

Finally, we validated the method by simulating a primary drug screening campaign. AmB (5 µg/mL) and non-treated wells were used as positive and negative inhibition controls, respectively. NIC (20 µM) served as a biofilm inhibitor to be identified during the screening. Briefly, 384-well plates were inoculated (1 × 10^6^ cells/mL in RPMI-1640), obtaining a final vol. of 50 µL/well. Plates were sealed with gas-permeable films and incubated at 37 °C for 24 h. Following incubation, plates were processed and stained with safranin as described before. OD_490_ readings were then used to calculate Z′ scores and the percentage of inhibition.

## 3. Results and Discussion

### 3.1. Optimization of Safranin-Staining Conditions for C. albicans Biofilm in a 96-Well Plate Assay

**Figure 1:**
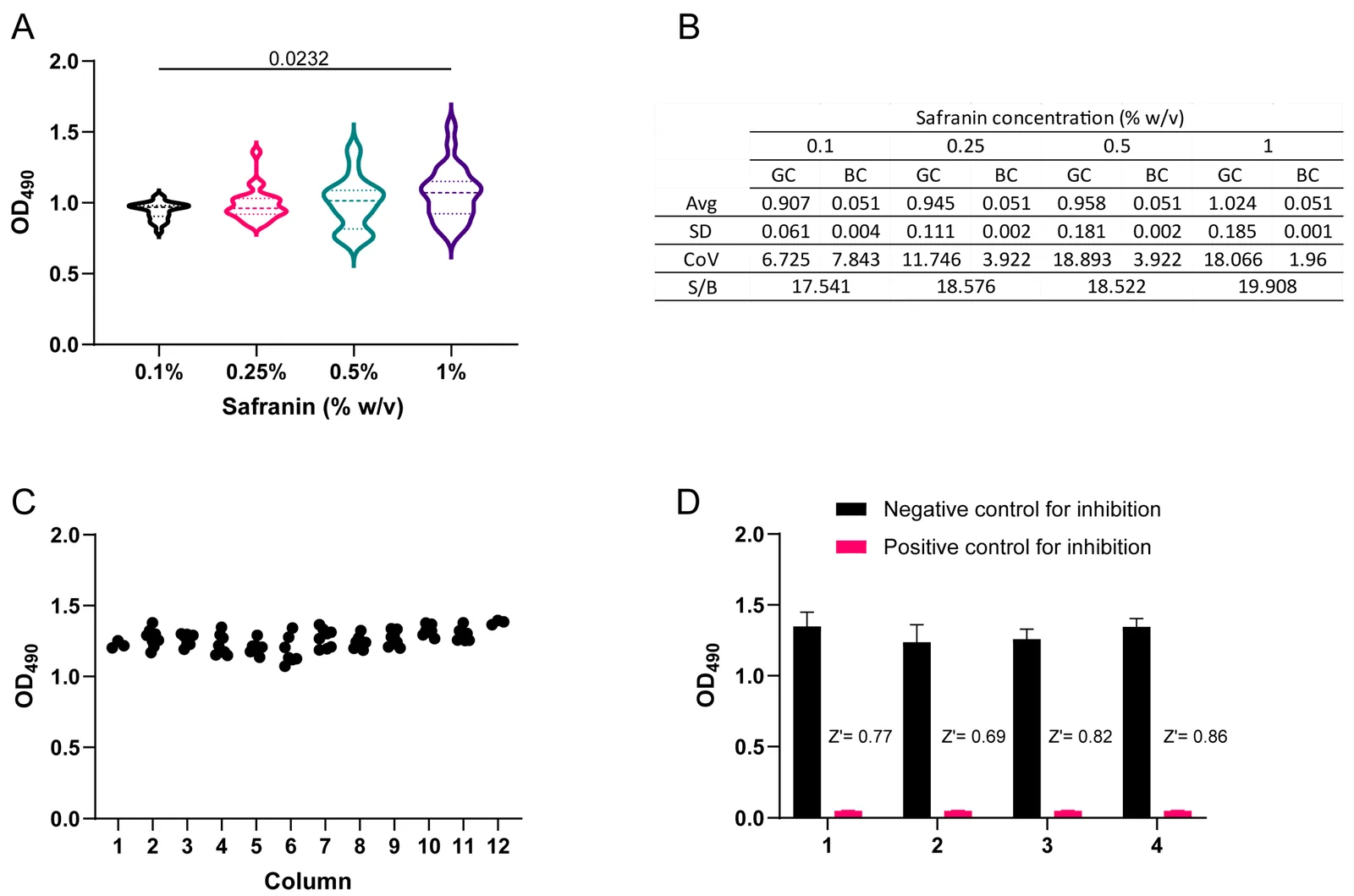
(**A**) Biofilms of *C. albicans* were stained with Safranin at various concentrations, and the staining capability was assessed by measuring the OD_490_. A one-way ANOVA with Tukey’s post hoc test was used to evaluate differences in staining efficiency at the different experimental steps. Only significant differences (*p* < 0.05) between treatments are displayed in the figure. (**B**) The average (Avg), standard deviation (SD), coefficient of variation (CoV), and signal-to-background ratio (S/B) were calculated for the corresponding growth control (GC) and background controls (BC) for each Safranin staining treatment. (**C**) Monitoring of OD_490_ signal uniformity from individual biofilms across a 96-well microtiter plate. (**D**) Evaluation of the assay quality of the Safranin-based well plate assay for large-scale screening of biofilm inhibitors for *C. albicans*. OD_490_ readings from positive control wells containing AmB at 05 µg/mL (red bar) and negative control wells with no drug (black bar). Z′-factor values were calculated from 4 independent biological experiments on 4 different days, obtaining values > 0.5 in all cases, indicating a high-quality assay.

To minimize the use of dye for biofilm staining and reduce potential waste, we evaluated different Safranin concentrations. As shown in Figure 1A, we first evaluated which Safranin concentration best stained *C. albicans* biofilms by measuring OD_490_ across treatments. A slight statistical difference (*p* = 0.023) was found between 0.1% and 1% Safranin, with mean OD_490_ values of 0.945 and 1.067, respectively. Standard deviation was higher with 1% (0.185) than with 0.1% (0.061), and the CoV was 17.43% vs. 6.4%, respectively. S/B ratios were similar across all treatments (Figure 1B). Due to minimal staining differences, lower variability, reduced toxicity, and lower cost, we selected a 0.1% Safranin concentration for subsequent experiments. We recommend preparing dilutions from a 1% (*w*/*v*) Safranin commercial stock to ensure consistency from batch to batch. The present staining procedure was specifically adapted for *C. albicans* SC5314, a well-characterized biofilm former and therefore the reference strain for antibiofilm drug screening campaigns. Due to differences in biofilm formation across *C. albicans* strains and *Candida* species, the biomass may stain differently, affecting direct OD_490_ readings. The Safranin staining procedure must be adapted and validated for different *Candida* strains and species, as some may be poorly suited to this protocol.

### 3.2. Safranin-Based 96-Well Plate Assay Demonstrated High Quality and Robustness for Drug Screening Applications

A high-quality and robust assay is crucial to ensure accurate identification of antibiofilm molecules and minimize variability in drug screening campaigns. We analyzed the uniformity of signals from individual Safranin-stained biofilms across the plate and calculated the Z′-factor to confirm the quality and robustness of the protocol [14]. Z′-factor values ranged from 0.69 to 0.86 across independent experiments conducted on different days, indicating adequate separation between the positive and negative controls (Figure 1C). Similarly, the raw signals were also distributed uniformly within each plate (Figure 1D). These findings support the applicability of the proposed protocol for large-scale screening under the experimental conditions evaluated [18].

In addition, it is important to note that our proposed safranin-based protocol was developed as an alternative method for screening chemical libraries for biofilm formation inhibition. The development and validation of this method do not intend to replace the “classical” Safranin staining method for purposes other than large-scale screening. Therefore, the results do not establish superiority or equivalence in sensitivity, dynamic range, reproducibility, or inter-batch variability. The main practical distinction of the proposed method is its simplified, extraction-free workflow, which avoids organic solvents, reduces hazardous waste, and eliminates additional transfer steps, plates, and specialized equipment such as chemical fume hoods. These features may make the method particularly useful for laboratories with limited infrastructure or financial resources. Future studies should directly compare both methods under identical experimental conditions.

### 3.3. The 96-Well Protocol Showed Reproducibility and Potential to Identify Known Antibiofilm Compounds in a Simulated Primary Drug Screening

**Figure 2:**
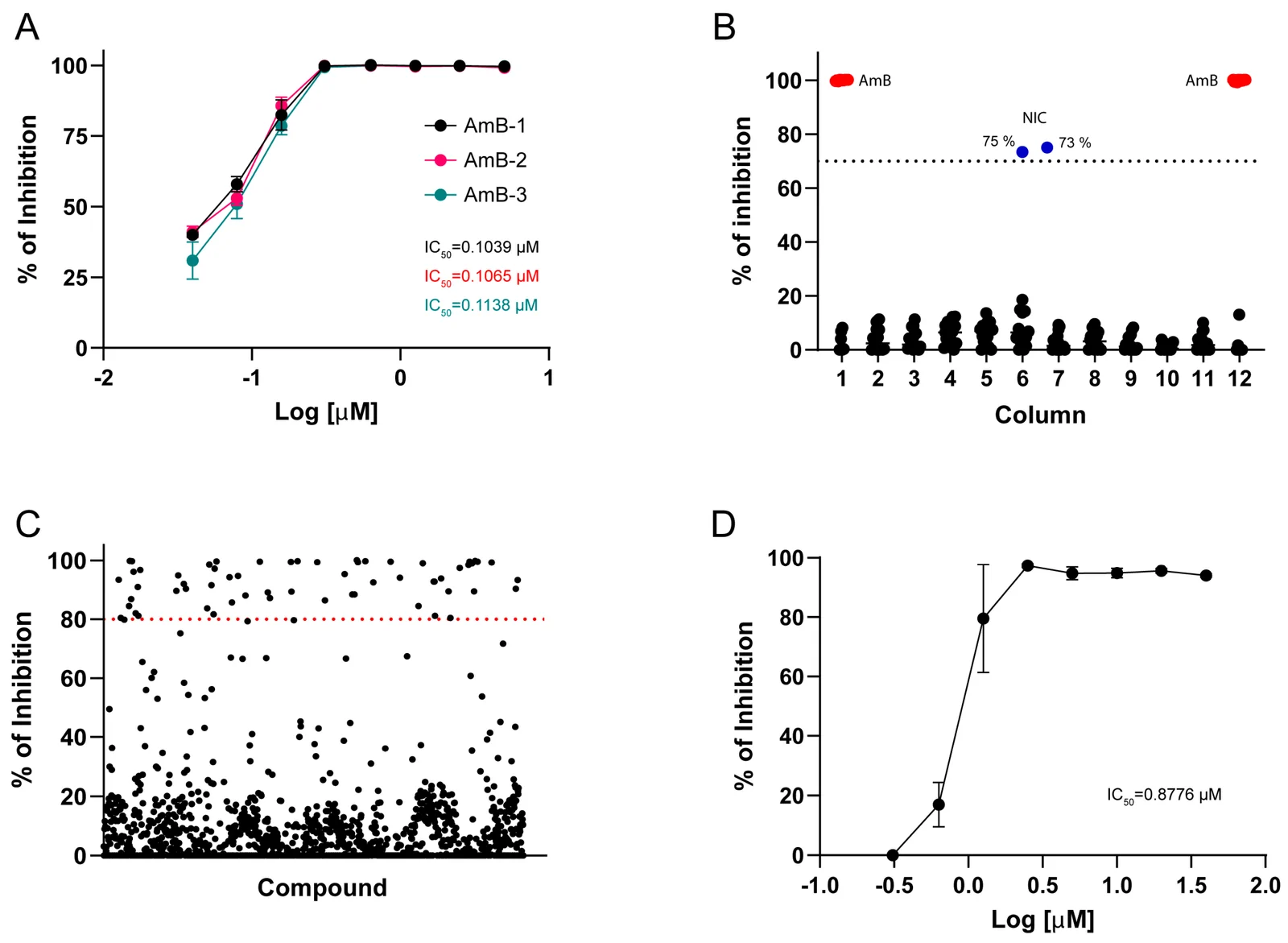
(**A**) Evaluation of the reproducibility of the Safranin-based well plate assay by dose–response experiments using amphotericin B (AmB). Different colors represent each biological replicate with its corresponding IC_50_ value. (**B**) Graphical representation of % inhibition data from the “simulated” primary screening performed in duplicate plates. Blue dots indicate the percentage inhibition value of Niclosamide (20 µM); red dots represent the positive controls for inhibition (AmB at 5 µg/mL), and black dots indicate non-treated biofilms. (**C**) Graphical representation of results from screening the Prestwick 1520 Chemical Library for inhibitors of biofilm formation against *C. albicans* using our proposed method. The dotted lines indicate the 80% arbitrary threshold for initial hit identification. (**D**) Confirmatory evaluation of the antibiofilm activity of Vinpocetine by dose–response experiments. The IC_50_ of Vinpocetine was calculated from 2 independent biological replicates with 6 technical replicates each.

As an additional reproducibility assessment, we performed dose–response experiments to confirm the activity of AmB and determine its potency. We experimentally determined the IC_50_ value for AmB from 3 biological replicates, obtaining values of 0.1039, 0.1138, and 0.1065 µM, and reproducible inhibition curves (Figure 2A). These results indicate that our protocol is highly reproducible and precise in assessing the inhibitory activity of potential antibiofilm agents under the evaluated conditions. The analytical precision relative to the classical Safranin method was not assessed. Moreover, the AmB inhibition curves generated with our protocol were similar to those obtained under the same experimental conditions using XTT to measure metabolic activity (Supplementary Figure S1). We then evaluated the ability of our protocol to identify a positive biofilm inhibitor (Niclosamide—NIC) in a simulated primary drug screening. From these experiments, NIC exhibited 73% and 75% inhibition in two independent experimental runs (Figure 2B). These results further support the suitability of this protocol for detecting potential hits in large-scale drug discovery campaigns targeting *Candida* biofilms.

### 3.4. Large-Scale Screening of the Prestwick Chemical Library to Identify Potential C. albicans Biofilm-Formation Inhibitors

We conducted a drug screening campaign by testing compounds from the Prestwick 1520 Chemical Library to identify potential antibiofilm agents. In this screening, hits were defined as compounds that inhibited biofilm formation by more than 80% (Figure 2C). Based on this criterion, 72 small molecules were identified (Supplementary Figure S2). Most of these compounds had been previously reported in previous *Candida albicans* screening campaigns [19,20,21,22], including our previous report on the screening of the Prestwick library [23], thereby further supporting the use of our screening method. Moreover, most of the 72 molecules have been reported to possess antifungal or antimicrobial activity; consequently, we opted to verify those lacking prior evidence of such activity. Following this criterion, we identified Vinpocetine, which, from our dose–response experiments, had a calculated IC_50_ of 0.8776 µM (Figure 2D). Vinpocetine is a synthetic derivative of vincamine, and it has been studied for its potential effects on blood flow and brain function [24,25,26]. It is often described as a nootropic or cognitive-support compound, and it has been marketed in some countries as a dietary supplement for memory, focus, and mental performance [27]. There are documented reports of extensive use of Vinpocetine as a dietary supplement, alongside previous preclinical and clinical studies detailing its toxicity and safety profile [28,29]. Mechanistic characterization of Vinpocetine to explore its antibiofilm and potential antifungal activities was excluded, as the main purpose of this study is to develop a practical protocol for screening thousands of compounds for biofilm formation inhibition. Notably, the calculated Z′-factor values for each plate used during this screening were all above 0.5, providing further reassurance of the utility of the methodology for large-scale screenings (Supplementary Figure S3).

### 3.5. Performance of the Miniaturized 384-Well Plate Assay and Identification of Known Biofilm Inhibitory Compounds in a Simulated Screening

**Figure 3:**
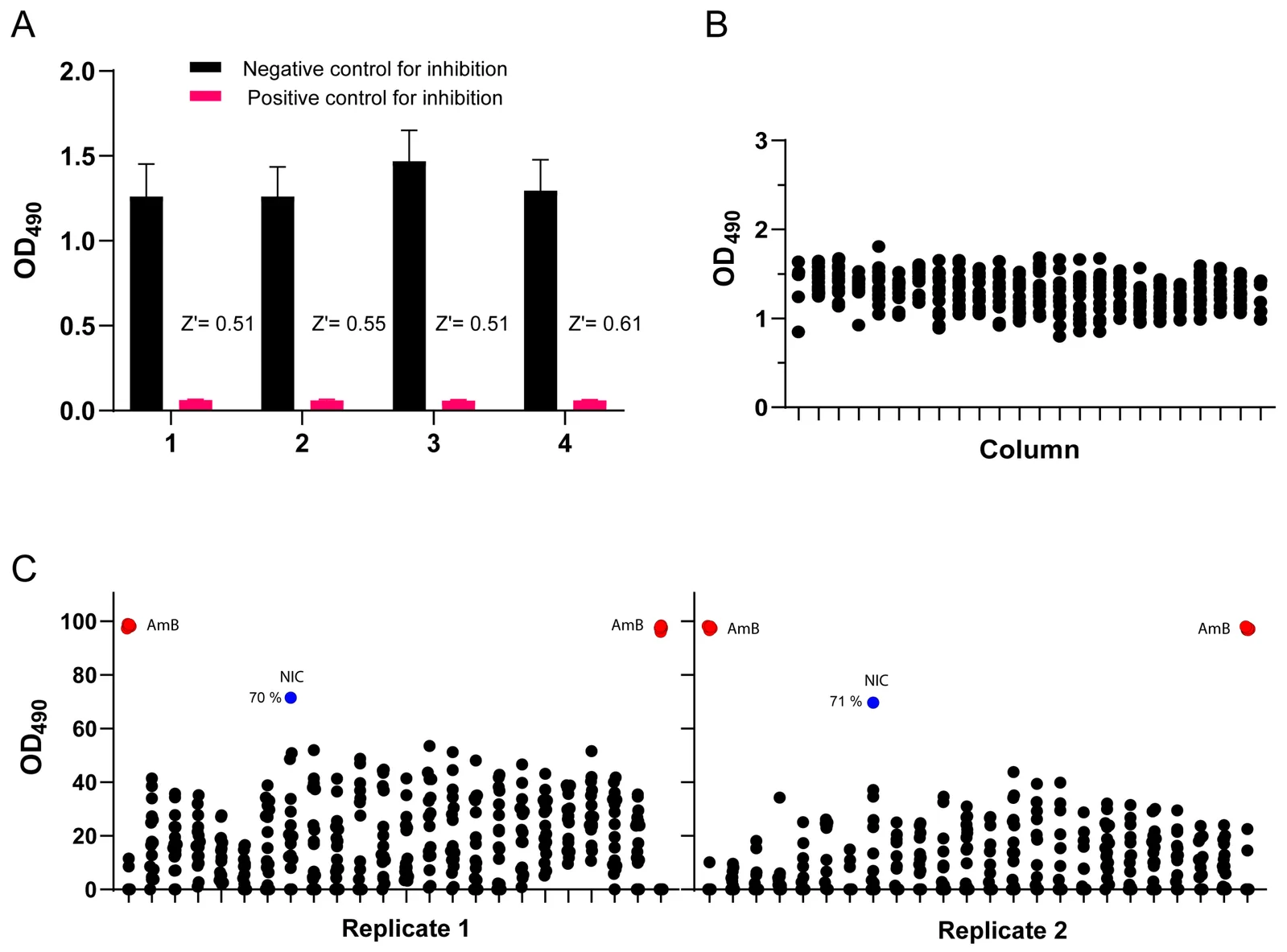
(**A**) OD_490_ signal uniformity across individual biofilms in a 384-well plate. (**B**) Robustness of the Safranin-based 384-well plate assay for large-scale screening of biofilm inhibitors for *C. albicans*. Positive control wells for inhibition (AmB at 05 µg/mL) (red bar) and negative control wells (black bar). Z′ values were calculated for each plate on independent days, showing values above 0.5 in all cases. (**C**) Graphical representation of % inhibition data from the “simulated” primary screening performed in duplicate plates. Blue dots indicate % inhibition of NIC (20 µM); red dots represent the positive controls for inhibition (AmB at 5 µg/mL), and black dots indicate non-treated biofilms.

After evaluating the 96-well plate method and screening the Prestwick Chemical Library, we adapted the protocol to a 384-well plate format for an initial assessment of its potential applicability, enabling true high-throughput screening. To determine the robustness and quality of the assay, negative (non-treated) and positive inhibition (AmB at 5 µg/mL) controls were included across independent experiments and used to calculate the Z′ factor. The 384-well plate assay produced Z′-factor values between 0.51 and 0.61, meeting the commonly used criterion used (Z′-factor > 0.5), showing sufficient separation between the positive and negative controls (Figure 3A). The signal distribution across the plate was uniform, confirming the quality of the assay [18] (Figure 3B). Finally, the method was further evaluated by performing a simulated primary screening to identify a known biofilm inhibitor (NIC at 20 µM). We were able to detect the positive inhibitor in 2 independent runs, obtaining inhibition percentages of 71 and 70, which were also consistent with our results using the 96-well plate format (Figure 3C). These preliminary results support further evaluation of the 384-well plate format for high-throughput screening of chemical libraries for the discovery of novel biofilm formation inhibitors.

## 4. Conclusions

In conclusion, we developed an alternative, simplified, extraction-free, and low-toxicity Safranin-based method to identify potential inhibitors of *C. albicans* biofilm formation. The protocol produced Z′-factors > 0.5 in both the 96-well and 384-well plate formats, supporting its application in large-scale screening. A total of 1520 molecules were screened using our 96-well plate protocol, identifying Vinpocetine as an interesting compound with biofilm-inhibiting activity that has not been previously reported for *Candida albicans* or any other fungal species. This Safranin screening protocol provides an accessible method to accelerate the identification of biofilm inhibitors across various laboratory settings, without the need for complex, costly instrumentation. This protocol is presented as an alternative method for screening chemical libraries and provides a simplified, extraction-free workflow under the experimental conditions evaluated in this study; it is not intended to claim superior analytical quality or performance. Orthogonal assays, including methods that measure metabolic activity, remain recommended. This protocol may be applicable to other *C. albicans* strains or *Candida* species; however, preliminary testing is advised to ensure optimal staining, uniform signal intensity, and minimized variability.

## 5. Limitations of the Protocol

Biofilm heat fixation is appropriate and allows for reproducible endpoint quantification of adherent Safranin-stained biofilm biomass in the current drug screening protocol; however, potential effects on matrix components or the native biofilm structure cannot be excluded. Future methodological studies could compare fixation procedures using microscopy and quantitative staining.

## Data Availability

No new data were created or analyzed in this study. Data sharing is not applicable to this article.

## Supplementary Materials

The following supporting information can be downloaded at https://www.mdpi.com/article/10.3390/jof12080581/s1, Figure S1: Assessment of the reproducibility of the Safranin-based well plate assay and the XTT-based assay was conducted using dose–response experiments with amphotericin B (AmB). Both assays were used to generate inhibition curves for *C. albicans* biofilm formation. Different colors represent each biological replicate; Figure S2: List of potential inhibitors of *C. albicans* biofilm formation identified in the primary drug screening of the Prestwick 1520 Chemical Library. Primary hits were defined as compounds that inhibited biofilm formation by more than 80%; Figure S3: Z′-factor values calculated for each plate used during the primary screening of the Prestwick 1520 library. As observed, all values across all plates are well above 0.5, indicating high quality, robustness, and effective signal-to-noise separation during screening.
