# Supplementary material for: Development of a Cost-Effective and Low-Toxicity Safranin-Based Assay for Discovering Candida albicans Biofilm Formation Inhibitors in Large-Scale Screening Campaigns
Source: J Fungi (Basel). 2026 Aug 7;12(8):581. doi: 10.3390/jof12080581 (PMC13514886; doi:10.3390/jof12080581)
Supplement: Supplementary file 1 [file jof-12-00581-s001.zip › jof-4457198-supplementary.pdf]

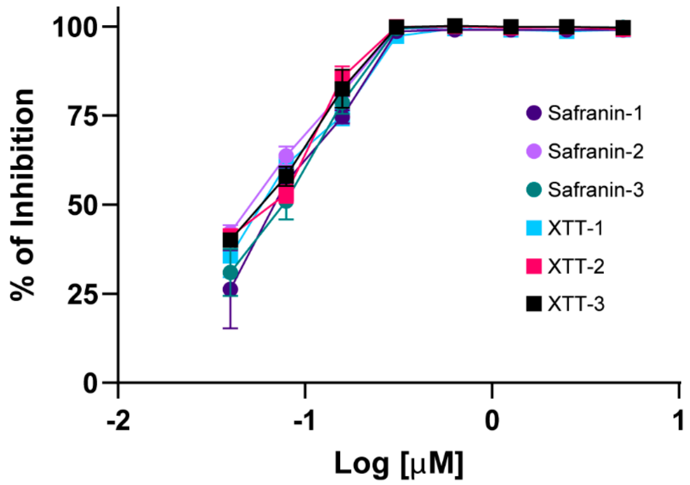

Supplementary Figure S1. Assessment of the reproducibility of the Safranin-based well plate assay and the XTT-based assay was conducted using dose-response experiments with amphotericin B (AmB). Both assays were used to generate inhibition curves for *C. albicans* biofilm formation. Different colors represent each biological replicate.

Compound	% of inhibition
Thonzonium bromide	100.05
Chloroxine	99.83
Thimerosal	99.76
Bacitracin	99.75
Chlorhexidine	99.74
Benzethonium chloride	99.73
Haloprogin	99.63
Broxyquinoline	99.56
Clindamycin Phosphate	99.56
Pyrvinium pamoate	99.54
Dithiazanine iodide	99.50
Methyl benzethonium chloride	99.49
Clioquinol	99.34
Alexidine dihydrochloride	99.32
Chlorquinaldol	99.30
Triclosan	99.00
Nitroxoline	98.98
Dequalinium dichloride	98.53
Cholecalciferol	98.49
Ebselen	97.42
Suloctidil	97.17
Isoconazole	96.75
Tioconazole	96.15
Sulconazole nitrate	95.32
Econazole nitrate	94.91
Ciclopirox ethanolamine	94.75
Butoconazole nitrate	94.27
Sertaconazole nitrate	94.13
Terbinafine	93.85
Miconazole	93.44
Lanoconazole	93.31
Bifonazole	92.80
Fluconazole	92.80
Enilconazole	92.50
Clotrimazole	92.04
Ketoconazole	91.53
Mefloquine hydrochloride	90.93
Vinpocetine	90.37
Salinomycin sodium	90.36
Trifluoperazine dihydrochloride	89.67
Amorolfine hydrochloride	89.45
Climbazole	89.43
Oxiconazole nitrate	89.36
Voriconazole	89.12
Flucytosine	88.39
Posaconazole hydrate	88.39
Xamoterol hemifumarate	88.39
Terconazole	88.09
Teriflunomide	87.18
Dehydrocholic acid	86.83
Auranofin	86.43
Amiodarone hydrochloride	85.67
Amfepramone hydrochloride	84.52
Aripiprazole	84.47
(Z,E) Clomiphene citrate	83.66
Perphenazine	82.04
Fusidic acid sodium salt	81.68
Hexachlorophene	81.19
Tamoxifen citrate	81.18
Isoxsuprine hydrochloride	80.47
Triclabendazole	80.47

Supplementary Figure S2. List of potential inhibitors of *C. albicans* biofilm formation identified on the primary drug screening of the Prestwick 1520 Chemical Library. Primary hits were defined as compounds that inhibited biofilm formation by more than 80%.

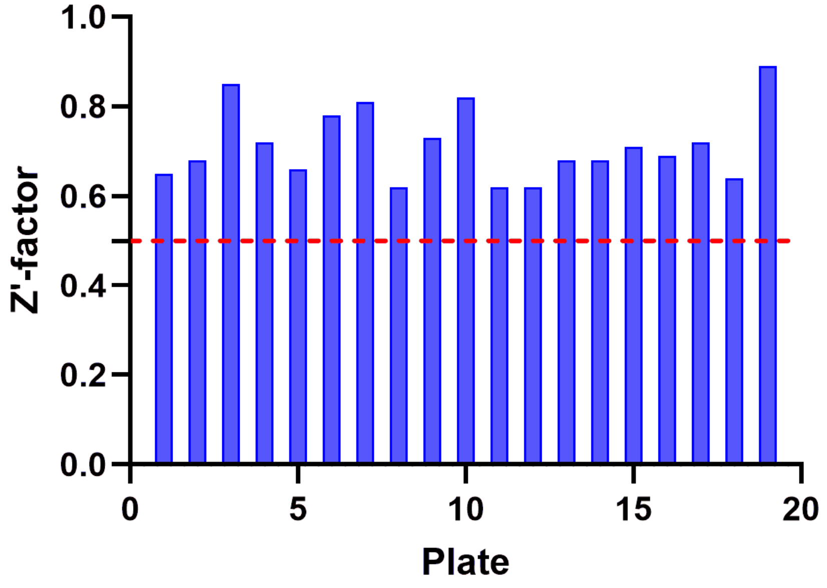

Supplementary Figure S3. Z'-factor values calculated for each plate used during the primary screening of the Prestwick 1520 library. As observed, all values across all plates are well above 0.5, indicating high quality, robustness, and effective signal-to-noise separation during screening.
